# Association of *MTHFD1* gene polymorphisms and maternal smoking with risk of congenital heart disease: a hospital-based case-control study

**DOI:** 10.1186/s12884-022-04419-2

**Published:** 2022-01-31

**Authors:** Xinli Song, Qiongxuan Li, Jingyi Diao, Jinqi Li, Yihuan Li, Senmao Zhang, Lijuan Zhao, Letao Chen, Jianhui Wei, Jing Shu, Yiping Liu, Mengting Sun, Peng Huang, Tingting Wang, Jiabi Qin

**Affiliations:** 1 Department of Epidemiology and Health Statistics, Xiangya School of Public Health, Central South University, 110 Xiangya Road, Changsha, 410078 Hunan China; 2 Guangdong Cardiovascular Institute, Guangdong Provincial People’s Hospital, Guangdong Academy of Medical Sciences, Guangzhou, Guangdong China; 3 NHC Key Laboratory of Birth Defect for Research and Prevention, Hunan Provincial Maternal and Child Health Care Hospital, 53 Xiangchun Road, Changsha, 410028 Hunan China; 4 Hunan Provincial Key Laboratory of clinical epidemiology, Changsha, Hunan China; 5 Department of Cardiothoracic Surgery, Hunan Children’s Hospital, Changsha, Hunan China

**Keywords:** Congenital heart disease, Smoking, *MTHFD1* gene, Interaction effects, Case-control study

## Abstract

**Background:**

*MTHFD1* gene may affect the embryonic development by elevated homocysteine levels, DNA synthesis and DNA methylation, but limited number of genetic variants of *MTHFD1* gene was focused on the association with congenital heart disease (CHD). This study examined the role of *MTHFD1* gene and maternal smoking on infant CHD risk, and investigated their interaction effects in Chinese populations.

**Methods:**

A case-control study of 464 mothers of CHD infants and 504 mothers of health controls was performed. The exposures of interest were maternal tobacco exposure, single nucleotide polymorphisms (SNPs) of maternal *MTHFD1* gene. The logistic regression model was used for accessing the strength of association.

**Results:**

Mothers exposed to secondhand smoke during 3 months before pregnancy (adjusted odds ratio [aOR] = 1.56; 95% confidence interval [CI]: 1.13–2.15) and in the first trimester of pregnancy (aOR = 2.24; 95%CI: 1.57–3.20) were observed an increased risk of CHD. Our study also found that polymorphisms of maternal *MTHFD1* gene at rs1950902 (AA vs. GG: aOR = 1.73, 95% CI: 1.01–2.97), rs2236222 (GG vs. AA: aOR = 2.38, 95% CI: 1.38–4.12), rs1256142 (GA vs.GG: aOR = 1.57, 95% CI: 1.01–2.45) and rs11849530 (GG vs. AA: aOR = 1.68, 95% CI: 1.02–2.77) were significantly associated with higher risk of CHD. However, we did not observe a significant association between maternal *MTHFD1* rs2236225 and offspring CHD risk. Furthermore, we found the different degrees of interaction effects between polymorphisms of the *MTHFD1* gene including rs1950902, rs2236222, rs1256142, rs11849530 and rs2236225, and maternal tobacco exposure.

**Conclusions:**

Maternal polymorphisms of *MTHFD1* gene, maternal tobacco exposure and their interactions are significantly associated with the risk of CHD in offspring in Han Chinese populations. However, more studies in different ethnic populations with a larger sample and prospective designs are required to confirm our findings.

**Trial registration:**

Registration number: ChiCTR1800016635.

**Supplementary Information:**

The online version contains supplementary material available at 10.1186/s12884-022-04419-2.

## Background

Congenital heart defect (CHD) was often defined as a structural or functional abnormality of the heart and/or great vessels that were present at birth [1]. Among all recognized structural birth defects, CHD was the most common and severe, with 4 to 10 cases per 1000 live births, which imposed a huge economic burden on the society and family [2]. Although the past few decades have seen a rapidly growing interest in exploring the etiology of CHD, the pathogenesis of most CHD cases remains unknown [3, 4].

So far, folic acid supplementation was the most effective large-scale primary intervention for decreasing CHD in China [5–9]. The folate-cycle product homocysteine might affect fetal heart development by disruption of gene methylation, increasing oxidative stress and homocysteinylation of key proteins [7, 10], which indicated that the occurrence of CHD was highly responsive to changes in genes related to maternal folate-homocysteine metabolism. The methylenetetrahydrofolate dehydrogenase 1 (*MTHFD1*) gene, located on chromosome 14q24, encoded the trifunctional enzyme MTHFD1 (5,10-methylenetetrahydrofolate dehydrogenase [11], 5,10-methenyltetrahydrofolate cyclohydrolase, and 10-formyltetrahydrofolate synthetase). This enzyme catalyzed three sequential reactions in the interconversion of tetrahydrofolate (THF) to 5,10-methylenetettrahydrofolate (5,10-methylene THF), the crucial substrate for 5-methyltetrahydrofolate (5-methlyTHF), which was required for DNA synthesis, DNA repair and provided the methyl donor for regeneration of methionine from homocysteine for subsequent methylation reactions [12, 13]. The data published indicated plausible mechanisms of the *MTHFD1* gene in CHD susceptibility might involve restricted DNA synthesis, high levels of homocysteine, and DNA methylation [7, 14–17]. The experimental studies had revealed that the *MTHFD1* gene with mutant genotypes expressed less stable in vitro and low active in vivo MTHFD1 protein [14, 15], and consequently disturbed de novo purine synthesis and impacted DNA synthesis. Moreover, the epidemiologic studies suggested that polymorphisms of the *MTHFD1* gene such as rs2236225 and rs1950902 were closely related to homocysteine levels [17–20] and DNA methylation [19]. *MTHFD1* R653Q (rs2236225 G → A) and *MTHFD1* R134K (rs1950902 G → A) as the two most well-studied polymorphisms [21], previous efforts involved in the associations of children’s genetic polymorphisms with CHD risk had yielded conflicting results [14, 22–27], and there were few studies on the associations of maternal genetic polymorphisms with CHD risk [14, 16]. Notably, previous studies focused mainly on a small number of functional nonsynonymous single-nucleotide polymorphisms (SNPs) of the *MTHFD1* gene with known biochemical phenotypes such as rs1950902 and rs2236225; the other significant variants have been largely ignored; thus this study represents both the first report and replication efforts in Han Chinese populations.

It had been reported that 85% of CHD resulted from a complex interplay of genetic variants and environmental factors [28]. Maternal tobacco exposure in the periconceptional period, defined as 3 months before pregnancy through the first trimester of pregnancy, was one of the most common environmental factors affecting abnormal fetal development. Amounts of epidemiologic studies about the associations of maternal tobacco exposure in the periconceptional period involved in smoke exposure and CHD risk were showed heterogeneous results, indicating that people had different susceptibility to the effects of tobacco exposure [29–33]. Convincing evidence showed that it was clear that particular genotypes of metabolizing systems and DNA repair pathways might modulate the effect leading to varying susceptibility to the CHD of tobacco exposure [34]. The maternal tobacco exposure was observed associations with substantial reductions of folate levels in plasma [35, 36] as well as in cord blood [37], and red blood cells [38, 39], even after correcting for folate intake [40], which implied that the interactive association between maternal tobacco exposure and gene polymorphisms in the folate-homocysteine pathway was closely related to CHD susceptibility. Although no direct evidence were reported about the interaction between maternal *MTHFD1* gene and smoking exposure on the CHD risk, some studies centered on 5,10-methylenetetrahydrofolate reductase (*MTHFR*) gene, an enzyme of great significance associated with folate metabolism, provided key clues. There were several studies in relation to the interaction of maternal *MTHFR* gene and smoke exposure on the risk of adverse pregnancy outcomes including low birth weight [41] and major birth defects such as neural tube defects, oral clefting [34] and CHD [42], and the mechanisms might be due to low folate status associated with genetic polymorphisms of *MTHFR* gene and smoke exposure [43]. MTHFD1 as a key enzyme catalyzed three sequential reactions in the interconversion of THF to 5,10-methylene THF, playing a significant role in the folate-homocysteine metabolism pathway, and thus we had reasons to believe *MTHFD1* gene also had an impact on folate status associated with smoke exposure. In addition, a recent study suggested that maternal folate levels might partly modify the influence of maternal tobacco exposure during pregnancy on the DNA methylation of the newborn epigenome and therefore affected embryonic development [44]. Many meaningful clues above were collected to put forward such a reasonable assumption that there existed the interaction effects of maternal tobacco exposure and the *MTHFD1* genetic variants, namely that the two factors jointly caused the CHD. In our study, a hospital-based case-control design based on the Han Chinese population was performed with the following objectives: (i) to assess the association of genetic variants of maternal *MTHFD1* gene with risk of CHD in offspring; (ii) to examine whether maternal smoking including active and passive smoking was significantly associated with risk of CHD in offspring; and (iii) to analyze the interaction effects between maternal smoking and the *MTHFD1* genetic variants for CHD.

## Methods

### Study design and recruitment of study participants

Recruitment was conducted by the Hunan Provincial Children’s Hospital (Changsha, Hunan Province, China). A total of 464 CHD patients and their mothers were consecutively enrolled from the Department of Cardiothoracic Surgery between November 2017 and December 2019. The non-CHD patients were from the Department of Child Healthcare in the same hospital during the same time period and were matched to the affected individuals by the age and sex of infants (frequency matching). The controls included 504 non-CHD patients who were without any congenital malformations after medical examination and their mothers. All CHD cases were diagnosed using echocardiography and confirmed by surgery. The CHD patients with structural malformations involving another organ system or known chromosomal abnormalities were excluded. Considering the homogenous ethnic background may reduce residual confounding factors from genetic and cultural differences, we only included the Han Chinese descent. We further excluded mothers who achieved pregnancy by assisted reproductive technology including in vitro fertilization (IVF) and intracytoplasmic sperm injection (ICSI) and reporting. Again, mothers who reported a history of depression or other psychiatric disorders or were diagnosed with depression or a psychiatric illness were also excluded. We classified the 464 CHD cases into 7 broad categories as described previously [45]. In particular, 28 (6.0%) had conotruncal defects, 360 (77.6%) had septation defects, 11 (2.4%) had left ventricular outflow tract obstruction, 17 (3.7%) had right ventricular outflow tract obstruction, 16 (3.4%) had anomalous pulmonary venous return, 20 (4.3%) had complex CHD, and 12 (2.6%) had other CHD defects (Table S1).

Our study was approved by the ethics committee of the Xiangya School of Public Health of Central South University, and written informed consent was obtained from all mothers. Besides, we have registered this study in the Chinese Clinical Trial Registry Center (registration number: ChiCTR1800016635).

### Information collection

The main exposures included maternal active smoking in 3 months before pregnancy, active smoking in the first trimester of pregnancy, passive smoking in 3 months before pregnancy and passive smoking in the first trimester of pregnancy. We defined maternal active smoking in 3 months before pregnancy and in the first trimester of pregnancy were mothers who reported active smoke during any of the 3 months before pregnancy, and during any of the first 3 months of pregnancy, respectively. The definitions of maternal passive smoking in 3 months before pregnancy and in the first trimester of pregnancy were mothers who reported that someone smoked at home and/or near her at work/school during any of the 3 months before pregnancy, and any of the first 3 months of pregnancy, respectively. A self-designed questionnaire was used to collect the corresponding information by specially trained investigators. This questionnaire was developed by experts in the field of CHD research and administered to eligible mothers (test-retest reliability = 0.833; Cronbach’s alpha = 0.782). Based on the previous literature [46–52], we selected the potentially confounding factors of significance as follows: maternal age at pregnancy onset (*< 35 or ≥ 35*), residence location (*rural or urban*), maternal education level (*≤9, 9–12, 12–16 or > 16 years*), annual income in the past 1 year (*≤50,000, 50,000–100,000, 100,000–150,000 or > 150000RMB*), history of adverse pregnancy outcomes (*yes or no*), consanguineous marriage (*yes or no*), history of congenital malformations in family (*yes or no*), cold or fever (*do you have a cold or fever experience in 3 months before pregnancy and/or during the first-trimester pregnancy, yes or no*), drinking alcohol (*do you have a drinking alcohol experience in 3 months before pregnancy and/or during the first-trimester pregnancy, yes or no*), drinking tea (*do you have a drinking tea experience in 3 months before pregnancy and/or during the first-trimester pregnancy, yes or no*), living near environmental pollution source (*yes or no*), dyeing hair or perming (*do you have a dyeing hair or perming experience in 3 months before pregnancy and/or during the first-trimester pregnancy, yes or no*), decorating housing (*are there any decorating housing at home or at work near you in 3 months before pregnancy and/or during the first-trimester pregnancy, yes or no*) and folate use (*do you use any folic acid in 3 months before pregnancy and/or during the first-trimester pregnancy, yes or no*). We defined folate use as any use of folic acid in 3 months before pregnancy and/or during the first-trimester pregnancy. The above mentioned information was further confirmed by consulting their Maternal and Child Health Manual and medical records. In China, each pregnant woman will be provided with a Maternal and Child Health Manual, which will record their basic demographic characteristics, behavioral habits, illness, and the results of various medical examinations during pregnancy. We also examined the SNPs of the maternal *MTHFD1* gene, which are described below.

### Sequencing of *MTHFD1* gene and genotyping

The *MTHFD1* gene was the candidate gene for the present study. When mothers completed the questionnaires mentioned above, they were asked to provide 3 to 5 ml of peripheral venous blood for genotyping. Methods for DNA extraction and genotyping have been described previously [40]. The polymorphisms of *MTHFD1* gene were tested using a matrix-assisted laser desorption and ionization time-of-flight mass spectrometry MassARRAY system (Agena iPLEX assay, San Diego, CA, USA). The laboratory technician, who performed the genotyping, retyped and double-checked each sample, and recorded the genotype data, was blinded to whether the samples were from cases or controls. SNP markers were selected using the SNPBrowser™ program (version 3.0) provided by AppliedBiosystems Inc. This program allowed the selection of SNP markers from the HapMap database. For each target gene, tagging SNPs were selected based on the pairwise r^2^ ≥ 0.8. However, we excluded these SNPs with minor allele frequencies less than 10% in Caucasians. We imposed a minimum SNP genotyping call rate at the level of 50%, which was applied to ensure data integrity of the individual’s genotypes that had been called. And successful rates for SNPlex assays were > 96% for 4 SNPs, from 90 to 96% for 1 SNPs. Finally, these genetic loci (rs1950902, rs2236225, rs2236222, rs11849530 and rs1256142) were selected as candidate loci for this study.

### Statistical analysis

Statistical analysis was performed using R software, version 3.5.0 (R Foundation for Statistical Computing). All tests were performed significantly for a two-sided *P* value not exceeding 0.05, except where otherwise specified. Additionally, the false discovery rate (FDR) control was used in this study to correct for multiple testing. The statistically significant results were those with the false discovery rate *P* value (FDR_*P*) < 0.1. Qualitative data were described using frequencies and percentages, and quantitative data were described using means and standard deviations (SDs). Hardy-Weinberg equilibrium (HWE) was tested for the control group (significance level at *P* < 0.01). We used a two-phase analytical method based on genetic model selection (GMS) to test associations between SNPs and CHD, and the specific calculation process had been described previously [53]. The genetic models contained the dominant model (calculated for mutant type homozygote versus wild type homozygotes and heterozygote), the recessive model (calculated for heterozygotes and mutant type homozygotes versus wild type homozygotes), and the additive model (calculated for wild type homozygotes versus heterozygote versus mutant type homozygote). We classified the genetic model into the recessive model if *Z*_HWDTT_ > c, the dominant model if *Z*_HWDTT_ < −c, and in the additive model if otherwise, where we chose c = Φ^− 1^(0.95) = 1.645. The Pearson χ^2^ test was used to compare the differences of nominal variables across groups. And for ordinal categorical variables, Wilcoxon rank sum test was used.

Odds ratios (OR) and their 95% confidence intervals (CI) were used to show the level of association. Univariate logistic regression analysis was done among all these potentially confounding factors mentioned above, and the variables with *p*-value < 0.05 were entered into the multivariable logistic regression as explanatory variables. There were no missing data. In initial analyses, the two variates maternal age at pregnancy onset and decorating housing did not show the statistically significant differences between the case and control group, so we finally included the following covariates as confounders of the association between maternal smoke exposure and *MTHFD1* gene on infant CHD risk in the multivariable model; residence location (rural or urban), maternal education level (≤9, 9–12, 12–16 or > 16 years), annual income in the past 1 year (≤50,000, 50,000–100,000, 100,000–150,000 or > 150000RMB), history of adverse pregnancy outcomes (yes or no), consanguineous marriage (yes or no), history of congenital malformations in family (yes or no), cold or fever (yes or no), drinking alcohol (yes or no), drinking tea (yes or no), living near environmental pollution source (yes or no), dyeing hair or perming (yes or no) and folate use (yes or no) were selected as confounding factors. The categorical explanatory variables with multiple categories were analyzed with indicator variables.

Gene-environment interactions were tested by introducing the multiplicative interaction term, all the covariates, and all the covariate-by-environment and the covariate-by-gene interaction terms into the same model, in order to properly control for potential confounders on the interaction term and assess its significance [54]. Of note, in the present study, we focused only on the risk of total CHD associated with maternal smoking and genetic variants of the *MTHFD1* gene and did not assess the risk of specific CHD subtypes due to the limited number of sample sizes for these subtypes.

## Results

### Baseline characteristics of study population

After considering the inclusion criteria, we finally recruited 464 CHD cases and their parents into the case group and 504 health infants and their parents into the control group. Comparisons of baseline characteristics across groups were summarized in Table 1. Our study showed that there were statistically significant differences between two groups for the following characteristics: residence location, maternal education level (years), annual income in the past 1 year (RMB), history of adverse pregnancy outcomes, consanguineous marriage, history of congenital malformations in family, cold or fever in the periconceptional period, and personal lifestyle and habit in the periconceptional period including drinking alcohol, drinking tea, living near environmental pollution source, dyeing hair or perming and folate use (all *P* values < 0.05). Thus, these factors were adjusted when accessing the association of maternal tobacco exposure, the genetic variants of the maternal *MTHFD1* gene, and their interactions with the risk of CHD in offspring.

**Table 1:** Baseline characteristics in case and control groups^a^

Baseline characteristics	Control group (*n* = 504)	Case group (*n* = 464)	Univariate analysis^d^
Age at pregnancy onset (years)			χ^2^ = 0.191; *P* = 0.662
< 35	434(86.1%)	404(87.1%)	
≥ 35	70(13.9%)	60(12.9%)	
Residence location			χ^2^ = 39.390; *P* < 0.001
Rural areas	276(54.8%)	344(74.1%)	
Urban areas	228(45.2%)	120(25.9%)	
Education level (years)			*Z* = 12.306; *P* < 0.001^b^
≤ 9	6(1.2%)	66(14.2%)	
9–12	100(19.8%)	190(40.9%)	
12–16	168(33.3%)	130(28.0%)	
> 16	230(45.6%)	78(16.8%)	
Annual income in the past 1 year (RMB)			*Z* = 15.946; *P* < 0.001^b^
≤ 50,000	144(28.6%)	372(80.2%)	
50,000-100,000	216(42.9%)	68(14.7%)	
100,000-150,000	46(9.1%)	10(2.2%)	
> 150,000	98(19.4%)	14(3.0%)	
History of adverse pregnancy outcomes			χ^2^ = 12.033; *P* = 0.001
No	280(55.6%)	206(44.4%)	
Yes	224(44.4%)	258(55.6%)	
Consanguineous marriage			χ^2^ = 14.480; *P* < 0.001
No	502(99.6%)	446(96.1%)	
Yes	2(0.4%)	18(3.9%)	
History of congenital heart disease in family			χ^2^ = 20.759; *P* < 0.001
No	500(99.2%)	436(94.0%)	
Yes	4(0.8%)	28(6.0%)	
Cold or fever^c^			χ^2^ = 16.513; *P* < 0.001
No	446(88.5%)	366(78.9%)	
Yes	58(11.5%)	98(21.1%)	
Drinking alcohol^c^			χ^2^ = 9.060; *P* = 0.003
No	468(92.9%)	404(87.1%)	
Yes	36(7.1%)	60(12.9%)	
Drinking tea^c^			χ^2^ = 9.257; *P* = 0.002
No	402(79.8%)	404(87.1%)	
Yes	102(20.2%)	60(12.9%)	
Living near environmental pollution source^c^			χ^2^ = 38.443; *P* < 0.001
No	470(93.3%)	370(79.7%)	
Yes	34(6.7%)	94(20.3%)	
Dyeing hair or perming^c^			χ^2^ = 12.532; *P* < 0.001
No	474(94.0%)	406(87.5%)	
Yes	30(6.0%)	58(12.5%)	
Folate^c^			χ^2^ = 23.917; *P* < 0.001
No	34(6.7%)	78(16.8%)	
Yes	470(93.3%)	386(83.2%)	
Decorating housing^c^			χ^2^ = 2.757; *P* = 0.097
No	462(91.7%)	438(94.4%)	
Yes	42(8.3%)	26(5.6%)	

^a^Data presented as number (percentage) unless otherwise indicated

^b^The Wilcoxon rank-sum test method was used; otherwise, the χ^2^ test was used

^c^The exposure occurred in the periconceptional period

^d^*P* < 0.05 was considered to indicate a statistically significant difference

### Maternal tobacco exposure and risk of CHD in offspring

Table 2 showed the association between maternal smoking and the risk of CHD in offspring. The prevalence rate of active smoking in 3 months before pregnancy in our controls (2.0%) was lower than the smoking rate among Chinese women in China Adult Tobacco Survey Report in 2015 (2.7%) [55]. None of the mothers in cases and controls reported active smoking in the first trimester of pregnancy. Mothers who reported active smoking in 3 months before pregnancy had an increased risk of CHD in offspring compared with the controls (*P* < 0.001), but this association was not independent of potential confounders (*P* = 0.052). After adjustment for baseline data, mothers exposed to secondhand smoke in 3 months before pregnancy were observed an increased risk of CHD in offspring (aOR = 1.56; 95% CI: 1.13–2.15). Additionally, the risk of CHD in offspring was significantly higher among mothers who were exposed to secondhand smoke in the first trimester of pregnancy (aOR = 2.24; 95% CI: 1.57–3.20).

**Table 2:** Maternal smoking and risk of congenital heart defects in offspring^a^

Exposure	Control group	Case group	Univariate logistic regression			Multivariable logistic regression^c^		
	(*n* = 504)	(*n* = 464)	cOR	95% CI	*P*	aOR^b^	95% CI	*P* ^c^
Active smoking in 3 months before pregnancy								
No	494 (98.0%)	432 (93.1%)	1.00			1.00		
Yes	10 (2.0%)	32 (6.9%)	3.66	1.78–7.53	< 0.001	2.37	0.99–5.65	0.052
Active smoking in the first trimester								
No	504 (100%)	464 (100%)	–	–	–	–		–
Yes	0 (0%)	0 (0%)	–	–	–	–		–
Passive smoking in 3 months before pregnancy								
No	316 (62.7%)	222 (47.8%)	1.00			1.00		
Yes	188 (37.3%)	242 (52.2%)	1.83	1.42–2.37	< 0.001	1.56	1.13–2.15	0.007
Passive smoking in the first trimester								
No	406 (80.6%)	274 (59.1%)	1.00			1.00		
Yes	98 (19.4%)	190 (40.9%)	2.87	2.15–3.83	< 0.001	2.24	1.57–3.20	< 0.001

Abbreviations: *CI* confidence interval; *cOR* crude odds ratio; *aOR* adjusted odds ratio

^a^Data presented as number (percentage) unless otherwise indicated

^b^Adjusted for residence location, maternal education level (years), annual income in the past 1 year (RMB), history of adverse pregnancy outcomes, consanguineous marriage, history of congenital malformations in family, cold or fever in the periconceptional period, and personal lifestyle and habit in the periconceptional period including drinking alcohol, drinking tea, living near environmental pollution source, dyeing hair or perming and folate use

^c^*P* < 0.05 was considered to indicate a statistically significant difference

### Genotypes frequencies of SNPs and the results of HWE tests and GMS

The genotype frequencies for each SNP of the maternal *MTHFD1* gene and the results of HWE tests were summarized in Table S2. The HWE tests showed that the genotype frequencies of the 5 SNPs of maternal *MTHFD1* gene in the control group were all within HWE (all *P* values > 0.01). The results of the GMS of each SNP were presented in Table S3. The genetic models of SNPs including rs1950902, rs2236225, rs2236222 and rs11849530 were all classified into the additive model since *Z*_HWDTT_ > − 1.645 and *Z*_HWDTT_ < 1.645. The genetic model of rs1256142 was classified into the dominant model because of *Z*_HWDTT_ < − 1.645. We initially ascertained the genetic models of overall SNPs of the maternal *MTHFD1* gene, which was used for accessing the association between each SNP and risk of CHD in offspring based on the corresponding genetic model.

### Genetic variants of maternal *MTHFD1* gene and risk of CHD in offspring

The association between each maternal SNP of the *MTHFD1* gene and the risk of CHD in the Han Chinese population was shown in Table 3. The univariate analyses suggested that there were statistically significant differences for the genetic variants at rs1950902 (AA vs. GG: *P* = 0.006; the additive model: *P* = 0.002), rs2236222 (GG vs. AA: *P* = 0.001; the additive model: *P* = 0.001) and rs1256142 (GA vs. GG: *P* = 0.035) between the case and control groups.

**Table 3:** *MTHFD1* genes in mothers and risk of congenital heart disease in offspring

SNPs	Univariate logistic regression			Multivariable logistic regression^c^			
	cOR	95% CI	*P*	aOR	95% CI	*P*	FDR*_P*^d^
rs1950902							
G/G	1.00		–	1.00		–	–
G/A	1.29	0.85–1.96	0.224	1.38	0.80–2.39	0.247	0.309
A/A	1.80	1.18–2.73	0.006	1.73	1.01–2.97	0.046	0.090
Additive^a^	1.36	1.12–1.64	0.002	1.30	1.02–1.65	0.025	0.090
rs2236225							
G/G	1.00		–	1.00		–	–
G/A	1.21	0.92–1.59	0.178	1.16	0.81–1.65	0.427	0.493
A/A	1.16	0.61–2.20	0.648	1.03	0.47–2.27	0.937	0.937
Additive	1.15	0.92–1.44	0.211	1.09	0.82–1.45	0.546	0.585
rs2236222							
A/A	1.00		–	1.00		–	–
G/A	1.27	0.97–1.66	0.087	1.27	0.96–1.67	0.096	0.144
G/G	2.50	1.46–4.29	0.001	2.38	1.38–4.12	0.002	0.015
Additive	1.42	1.16–1.75	0.001	1.40	1.14–1.73	0.002	0.015
rs11849530							
A/A	1.00		–	1.00		–	–
G/A	0.91	0.69–1.20	0.498	1.24	0.87–1.77	0.243	0.309
G/G	1.13	0.77–1.65	0.536	1.68	1.02–2.77	0.042	0.090
Additive	1.02	0.86–1.22	0.809	1.28	1.02–1.62	0.037	0.090
rs1256142							
G/G	1.00		–	1.00		–	–
G/A	1.44	1.03–2.02	0.035	1.57	1.01–2.45	0.048	0.090
A/A	1.20	0.83–1.74	0.340	1.57	0.97–2.56	0.068	0.113
Dominant^b^	0.92	0.70–1.21	0.550	1.57	1.03–2.40	0.037	0.090

Abbreviations: *CI* confidence interval; *cOR* crude odds ratio; *aOR* adjusted odds ratio; *SNPs* single nucleotide polymorphisms; *MTHFD1* methylenetetrahydrofolate dehydrogenase 1; *FDR_P* false discovery rate *P* value

^a^Additive means wild type homozygotes vs. heterozygote vs. mutant type homozygote

^b^Dominant means wild type homozygote vs. mutant type homozygotes and heterozygote

^c^Adjusted for residence location, maternal education level (years), annual income in the past 1 year (RMB), history of adverse pregnancy outcomes, consanguineous marriage, history of congenital malformations in family, cold or fever in the periconceptional period, and personal lifestyle and habit in the periconceptional period including drinking alcohol, drinking tea, living near environmental pollution source, dyeing hair or perming and folate use

^d^FDR_*P* < 0.1 was considered to indicate a statistically significant difference

After adjustment for the potential confounders, the polymorphisms including rs1950902 (AA vs. GG: aOR = 1.73, 95% CI: 1.01–2.97; the additive model: aOR =1.30, 95% CI: 1.02–1.65), rs2236222 (GG vs. AA: aOR = 2.38, 95% CI: 1.38–4.12; the additive model: aOR = 1.40, 95% CI: 1.14–1.73) and rs1256142 (GA vs.GG: aOR = 1.57, 95% CI: 1.01–2.45; the dominant model: aOR = 1.57, 95% CI: 1.03–2.40) were still observed an increased risk for CHD, respectively. In addition, rs11849530 (GG vs. AA: aOR = 1.68, 95% CI: 1.02–2.77; the additive model: aOR = 1.28, 95% CI: 1.02–1.62) was also observed a significant association with higher CHD risk.

### Interactions between maternal smoke exposure and *MTHFD1* gene for risk of CHD

The gene-environment interaction effects between maternal SNPs of *MTHFD1* gene including rs1950902, rs2236222, rs1256142, rs11849530 and rs2236225 in the corresponding genetic model and maternal smoke exposure on CHD risk were summarized in Table 4. Due to the limited sample size of mothers who had an active smoke experience during the periconceptional period, the multivariable logistic regression models failed when adjusted for confounders, so we only explored the interactive effects of maternal passive smoke before pregnancy and in the first trimester, and *MTHFD1* gene on infant CHD risk. Our results showed there were statistically significant interaction effects between maternal passive smoking in 3 months before pregnancy and genetic variants of *MTHFD1* gene at rs2236225 (FDR_*P =* 0.003), rs2236222 (FDR_*P* < 0.001) and rs1256142 (FDR_*P* < 0.001). Additionally, maternal passive smoking in the first trimester was also observed statistically significant interaction effects with *MTHFD1* gene at rs2236225 (FDR_*P =* 0.043), rs2236222 (FDR_*P =* 0.035) and rs1256142 (FDR_*P =* 0.035).

**Table 4:** Interactions between SNPs of *MTHFD1* gene and maternal smoking detected by logistic regression

SNPs	Passive smoking before pregnancy			Passive smoking in the first trimester		
	aOR (95%CI)	*P*	FDR*_P*	aOR (95%CI)	*P*	FDR*_P*
rs1950902 (additive)	1.09(0.49–2.45)	0.830	0.830	1.13(0.44–2.91)	0.800	0.800
rs2236225 (additive)	3.65(1.62–8.22)	0.002	0.003	3.17(1.15–8.75)	0.026	0.043
rs2236222 (additive)	0.32(0.18–0.60)	< 0.001	< 0.001	0.43(0.23–0.83)	0.011	0.035
rs11849530 (additive)	1.28(0.58–2.82)	0.538	0.673	1.82(0.72–4.60)	0.207	0.259
rs1256142 (dominant)	0.24(0.12–0.45)	< 0.001	< 0.001	0.45(0.24–0.85)	0.014	0.035

Abbreviations: *aOR* adjusted odds ratio; *CI* confidence interval; *SNPs* single nucleotide polymorphisms; *MTHFD1* methylenetetrahydrofolate dehydrogenase 1; *FDR*_*P* false discovery rate *P* value

^a^Adjusted for residence location, maternal education level (years), annual income in the past 1 year (RMB), history of adverse pregnancy outcomes, consanguineous marriage, history of congenital malformations in family, cold or fever in the periconceptional period, and personal lifestyle and habit in the periconceptional period including drinking alcohol, drinking tea, living near environmental pollution source, dyeing hair or perming and folate use

^b^FDR_*P* < 0.1 was considered to indicate a statistically significant difference

Crossover analysis was conducted in order to further elaborate the gene-environment interaction effects of maternal passive smoking in 3 months before pregnancy (Table S4) and in the first trimester (Table S5), and maternal *MTHFD1* gene on the risk of CHD. Mothers with the wild type genotype meanwhile without passive smoke exposure were considered to be the reference group. After the adjustment for confounders, it was suggested that mothers who had variant genotypes at rs2236225 (aOR = 1.96; 95% CI: 1.19 to 3.23; FDR_*P =* 0.034), rs11849530 (aOR = 2.01; 95% CI: 1.25 to 3.22; FDR_*P =* 0.020) and rs1256142 (aOR = 2.94; 95% CI: 1.54 to 5.62; FDR_*P =* 0.008), and meanwhile were exposed to second smoke before pregnancy were at a significantly higher risk of CHD in offspring compared with those in the reference group (Table S4). We also observed that mothers who exposed to second smoke in the first trimester, and meanwhile had variant genotypes at rs2236225 (aOR = 2.93; 95% CI: 1.64 to 5.24; FDR_*P* < 0.001), rs2236222 (aOR = 1.85; 95% CI: 1.05 to 3.27; FDR_*P =* 0.069), rs11849530 (aOR = 3.22; 95% CI: 1.91 to 5.45; FDR_*P* < 0.001) and rs1256142 (aOR = 3.36; 95% CI: 1.88 to 5.99; FDR_*P* < 0.001) were at a significantly higher risk of CHD in offspring compared with those in the reference group (Table S5).

## Discussion

Convincing evidence implies that periconceptional intake of folic acid leads to a 40 to 60% reduction in the risk of a CHD-affected delivery, which makes investigating the association between genetic polymorphisms in genes related to folate metabolism and CHD risk an attractive pursuit [56]. The *MTHFD1* gene plays a key role in the folate-homocysteine metabolism pathway, encoding a single protein with three catalytic properties crucial for DNA synthesis, DNA repair, and methylation reactions. The epidemiologic and experimental studies indicate the plausible association between genetic variants of the *MTHFD1* gene and the risk of CHD [14–18], but previous efforts on the association have yielded controversial results, with the majority limited to a small number of functional nonsynonymous polymorphisms [14, 20, 22]. This is therefore the first study to explore the other variants of the *MTHFD1* gene on CHD risk in Han Chinese populations. Furthermore, folate-homocysteine metabolism may partly modulate the effect leading to varying susceptibility to the CHD of tobacco exposure [34, 42–44]. Thus this study also seeks to examine the interactions of maternal tobacco exposure and polymorphisms of the *MTHFD1* gene on CHD risk, which may help to provide new clues for future etiological research and intervention of CHD.

In the present study, four genetic variants of maternal *MTHFD1* gene including rs1950902, rs2236222, rs1256142 and rs11849530 were revealed to have significant associations with an increased risk of CHD in case-control studies based on the Han Chinese population. The information from the public databases and the literature revealed unequivocally these SNPs were all within the coding region; one was functional nonsynonymous polymorphism with a known biochemical phenotype and the other were intronic ones [11, 14]. Notably, the well-studied polymorphism rs1950902 G → A (*MTHFD1* G401A) leads to an arginine (G allele) to a lysine (A allele) substitution, lying within the dehydrogenase/cyclohydrolase domain of *MTHFD1* protein. Although its role is unclear, it was speculated that the mutant genotypes of the *MTHFD1* gene at rs1950902 may influence the stability of the enzyme and change the catalytic activity [20], causing disturbance of the folate-homocysteine metabolism pathway. It was reported that rs1950902 was significantly related to elevated plasma homocysteine and reduced folate levels [18, 19]. These observations suggested that *MTHFD1* rs1950902 could affect embryonic development employing restricted DNA synthesis and a high level of homocysteine. Considering the results of the present study and the phenotypes related to rs1950902, we postulate that this polymorphism plays a role in fetal heart development. One previous study [24], however, found no evidence of a significant association between children’s *MTHFD1* 1,950,902 and Tetralogy of Fallot. Due to our limited sample size of each subtype of CHD cases, we didn’t analyze the association between specific CHD subtypes and genetic variants of maternal *MTHFD1* gene. Besides, the present study modestly found the intronic polymorphisms including rs2236222, rs1256142 and rs11849530 were associated with increased CHD risk, and the plausible mechanism of these polymorphisms increasing CHD susceptibility was likely to affect codon usage and translational efficiency [57].

Particularly, it was worth mentioning that *MTHFD1* rs2236225 (*MTHFD1* R653Q), the most investigated genetic variant of the *MTHFD1* gene, leads to an arginine (G allele) to glutamine (A allele) substitution in the MTHFD1 protein. Convincing evidence suggested that mutant type protein of *MTHFD1* gene at rs2236225 caused significant DNA synthesis restriction [14] and increased homocysteine levels [17–19]. In addition, the specific CHD subtypes such as atrial septal defects [16], tetralogy of Fallot [58] and aortic stenosis [14] had been reported significant associations with children’ rs2236225, but previous efforts involved in their associations of children’ rs2236225 and CHD risk had yielded conflicting [14, 20, 22]. For the maternal *MTHFD1* rs2236225, one previous study observed that maternal rs2236225 was associated with offspring CHD risk, and that rs2236225 was related to lower serum folic acid levels and higher homocysteine levels. However, neither Christensen KE’s nor our data revealed a significant association between maternal rs2236225 and offspring CHD risk. These inconclusive findings might result from the genetic differences between different populations of people, and might also partly be explained by the different susceptibility of folate-homocysteine imbalance to every subtype of CHD [7]. Evidence suggested that conotruncal defects and ventricular septal defects likely shared some etiologic and specifically genetic risk factors, and both were likely associated with disruption of the development of the cardiac neural crest, which was highly responsive to folate [7]. Overall, some of the polymorphisms involved in our study had not been confirmed before, and literature involved in the association between genetic variants of maternal *MTHFD1* gene and CHD was still lack. It needs further and clearer evidence to figure out the mechanism.

We also examined the association between maternal smoking including active and passive smoking and the risk of CHD in offspring. Findings from the present study suggested that mothers who reported passive smoking at home or in the workplace 3 months before pregnancy and the first trimester of pregnancy were observed a 1.56-fold and 2.24-fold increased CHD risk, respectively, which was basically consistent with a recent meta-analysis [59]. Obviously, the maternal passive tobacco exposure in the first trimester of pregnancy was shown more harmful than in 3 months before pregnancy, which may be partly explained by the fact that the former was the sensitive period for fetal heart development. Additionally, a great number of previous epidemiologic studies supported that periconceptional active smoking was associated with risk of CHD in offspring [59]. However, we modestly did not observe that maternal active smoking in 3 months before pregnancy could increase CHD risk after adjusted potential confounders, and even none of our subjects reported active smoking during pregnancy. The causes for the insignificant association between maternal active smoking in 3 months before pregnancy and CHD risk may be due to our limited sample size and the subjective smoking records. There was a possibility that pregnant smokers underreported their smoking and such potential misclassification might lead to underestimation of the impact of maternal active smoking on CHD. The behavior was attributed to medical and societal pressures that made pregnant women reluctant to report their smoking activities [60].

Our results also showed the different degrees of interaction effects between polymorphisms of *MTHFD1* gene including rs1950902, rs2236222, rs1256142, rs11849530 and rs2236225 and maternal passive smoking. However, studies concerning the interactions of SNPs in folate-related genes and maternal tobacco exposure were lack, Hobbs [42] indicated that the combined effect of elevations in maternal homocysteine, smoking, and the *MTHFR* CC genotype increased the risk of having a CHD-affected pregnancy (aOR = 11.8), compared with women who did not smoke, did not have an elevated homocysteine, and had the *MTHFR* 677 CC genotype served as the reference group. Possible mechanisms by which the *MTHFD1* gene interacted with maternal tobacco exposure to increase CHD susceptibility include elevated serum homocysteine, increased DNA methylation, and DNA synthesis restriction. It was well-studied that the maternal tobacco exposure was observed associations with substantial reductions of folate levels [35–37, 40], elevated levels of homocysteine [61] in cord blood, and altered global genomic methylation. The polymorphisms of the *MTHFD1* gene also showed a close relation to plasma homocysteine and DNA methylation. Hence, the mutant type of MTHFD1 protein and periconceptional tobacco exposure jointly caused the plasma homocysteine elevated, and high levels of homocysteine consequently affected fetal heart development by disruption of gene methylation, increasing oxidative stress and homocysteinylation of key proteins. Additionally, a recent study suggested that maternal folate-homocysteine metabolism may partly modify the influence of maternal tobacco exposure during pregnancy on the DNA methylation of newborn epigenome and therefore affected embryonic development [44], which provided indirect evidence to support our findings. Nevertheless, specific mechanisms were unclear and needed further research.

The limitations of the study need to be addressed. First, based on a case-control study, information on maternal tobacco exposure was collected through self-reported interviews, and therefore recall bias inevitably had to be taken into consideration. To reduce recall bias to some extent, the exposure information was further confirmed by consulting their Maternal and Child Health Manual and medical records. Second, despite adjusting many confounders, potential confounding factors cannot be entirely ruled out. Third, there are so many key enzyme genes in relation to folate metabolism pathway that are also involved in cardiovascular development, such as *MTHFR*, *MTRR* and *MTR* gene, but we only focused on the association between maternal *MTHFD1* gene and offspring CHD risk*.* Fourth, even if the analyzed SNP is located in the *MTHFD1* gene region, there may be SNPs in linkage disequilibrium in other gene regions, and the possibility that the SNPs are related to the function of the other gene cannot be denied. Fifth, considering population stratification bias in epidemiologic studies, we recruited the participants restricted to the Han Chinese ethnicity, and further work was needed to estimate the effect of the *MTHFD1* gene and maternal tobacco exposure in CHD risk within other populations. Last, sample size limitations prevented us from examining specific CHD subtypes.

## Conclusions

The current findings observed that maternal polymorphisms of the *MTHFD1* gene at rs1950902, rs2236222, rs11849530, and rs1256142 were significantly associated with the risk of CHD in offspring. In addition, a positive association between maternal passive smoking in the periconceptional period and risk of CHD was found. Furthermore, our results showed the different degrees of interaction effects between polymorphisms of the *MTHFD1* gene including rs1950902, rs2236222, rs1256142, rs11849530 and rs2236225, and maternal tobacco exposure. However, considering the complexity of the mechanism and the limitation of sample size, more studies in different ethnic populations with a larger sample and prospective designs were required to confirm our findings.

## Supplementary Information

**Additional file 1.**

## Data Availability

The datasets supporting the conclusions of this article are included within the article and its additional files.

## Abbreviations

CHD: Congenital heart disease
aOR: Adjusted odds ratio
CI: Confidence interval
SNPs: Single nucleotide polymorphisms
*MTHFD1*: Methylenetetrahydrofolate dehydrogenase 1
FDR_*P*: False discovery rate *P* value
GMS: Genetic model selection
HWE: Hardy-Weinberg equilibrium
