# Supplementary material for: Association of MTHFD1 gene polymorphisms and maternal smoking with risk of congenital heart disease: a hospital-based case-control study
Source: BMC Pregnancy Childbirth. 2022 Jan 31;22:88. doi: 10.1186/s12884-022-04419-2 (PMC8805321; doi:10.1186/s12884-022-04419-2)
Supplement: Supplementary file 1 — Additional file 1. [file 12884_2022_4419_MOESM1_ESM.docx]

**Table S1. Classification of congenital heart diseases in cases**

| **CHD classification** | **Cases** | |
| --- | --- | --- |
|  | **No.** | **%** |
| Conotruncal defects | 28 | 6.0 |
| Tetralogy of Follot | 26 | 5.6 |
| Complete transposition of great arteries | 2 | 0.4 |
| Septation defects | 360 | 77.6 |
| Atrial septal defect | 66 | 14.2 |
| Ventricular septal defect | 252 | 54.3 |
| Atrioventricular septal defect | 42 | 9.1 |
| Left ventricular outflow tract obstruction | 11 | 2.4 |
| Right ventricular outflow tract obstruction | 17 | 3.7 |
| Anomalous pulmonary venous return | 16 | 3.4 |
| Complex CHDs | 20 | 4.3 |
| Other CHDs | 12 | 2.6 |

Abbreviations: CHDs = congenital heart diseases

**Table S2. Genotypes Frequencies of SNPs and *P* values of** **Hardy-Weinberg Disquilibrium test**

| **SNPs Group** | | **Genotype Frequencies** | | | **HWE test *P*** |
| --- | --- | --- | --- | --- | --- |
|  |  | **AA** | **AB** | **BB^‡^** |  |
| rs1950902 | Controls | 72(14.3%) | 242(48.0%) | 190(37.7%) | 0.719 |
|  | Case | 46(9.9%) | 200(43.1%) | 218(47.0%) |  |
| rs2236225 | Controls | 332(65.9%) | 152(30.2%) | 20(4.0%) | 0.620 |
|  | Case | 286(61.6%) | 158(34.1%) | 20(4.3%) |  |
| rs2236222 | Controls | 318(63.1%) | 164(32.5%) | 22(4.4%) | 0.883 |
|  | Case | 254(54.7%) | 166(35.8%) | 44(9.5%) |  |
| rs11849530 | Controls | 230(45.6%) | 206(40.9%) | 68(13.5%) | 0.047 |
|  | Case | 216(46.6%) | 176(37.9%) | 72(15.5%) |  |
| rs1256142 | Controls | 108(21.4%) | 246(48.8%) | 150(29.8%) | 0.703 |
|  | Case | 78(16.8%) | 256(55.2%) | 130(28.0%) |  |

Abbreviations: SNPs = single nucleotide polymorphisms; HWE = Hardy-Weinberg equilibrium.

^‡^ AA, homozygous wild-type;

AB, heterozygous variant;

BB, homozygous variant.

HWE was tested for the control group (significance level at *P* <0.01).

**Table S3. The genetic model selection of SNPs at *MTHFD1* gene**

| **SNPs** | **Genotypes** | **Control (n)** | **Case (n)** | ***Z*_HWDTT_^\|\|^** | **GMS** |
| --- | --- | --- | --- | --- | --- |
| rs1950902 | GG/GA/AA | 72/242/190 | 46/200/218 | 0.267 | additive |
| rs2236225 | GG/GA/AA | 332/152/20 | 286/158/20 | -0.562 | additive |
| rs2236222 | AA/GA/GG | 318/164/22 | 254/166/44 | 1.612 | additive |
| rs11849530 | AA/GA/GG | 230/206/68 | 216/176/72 | 1.137 | additive |
| rs1256142 | GG/GA/AA | 108/246/150 | 78/256/130 | -2.085 | dominant |

Abbreviations: SNPs = single nucleotide polymorphisms; *MTHFD1* = methylenetetrahydrofolate dehydrogenase 1; GMS = genetic model selection.

^||^We classified the genetic model into the recessive model if *Z*_HWDTT_ > c, the dominant model if *Z*_HWDTT_ < -c, and in the additive model if otherwise, where we chose c = Φ^-1^(0.95) = 1.645.

**Table S4. Crossover analysis in assessing the interactive effects between the maternal polymorphisms of *MTHFD1* gene and passive smoking before pregnancy on the risk of CHD**

| SNPs | Passive smoking before pregnancy | No. of  Control | No. of  Case |  | Multivariate analysis | |
| --- | --- | --- | --- | --- | --- | --- |
|  |  |  |  |  | aOR (95%CI)^b^ | FDR_*P*^a^ |
| rs1950902 |  |  |  |  |  |  |
| Wild genotype (GG) | No | 42 | 20 |  | 1.00 (reference) |  |
| Variant genotypes (GA+AA) | No | 274 | 202 |  | 1.38 (0.66 to 2.89) | 0.399 |
| Wild genotype (GG) | Yes | 30 | 26 |  | 1.24 (0.47 to 3.26) | 0.659 |
| Variant genotypes (GA+AA) | Yes | 158 | 216 |  | 2.14 (1.01 to 4.53) | 0.048 |
| *P*_interaction_ |  |  |  |  |  | 0.830 |
| rs2236225 |  |  |  |  |  |  |
| Wild genotype (GG) | No | 198 | 148 |  | 1.00 (reference) |  |
| Variant genotypes (GA+AA) | No | 118 | 74 |  | 0.83 (0.52 to 1.33) | 0.529 |
| Wild genotype (GG) | Yes | 134 | 138 |  | 1.17 (0.78 to 1.76) | 0.529 |
| Variant genotypes (GA+AA) | Yes | 54 | 104 |  | 1.96 (1.19 to 3.23) | 0.034 |
| *P*_interaction_ |  |  |  |  |  | 0.002 |
| rs2236222 |  |  |  |  |  |  |
| Wild genotype (AA) | No | 202 | 104 |  | 1.00 (reference) |  |
| Variant genotypes (GA+GG) | No | 114 | 118 |  | 1.59 (1.01 to 2.51) | 0.099 |
| Wild genotype (AA) | Yes | 116 | 150 |  | 2.07 (1.33 to 3.20) | 0.008 |
| Variant genotypes (GA+GG) | Yes | 72 | 92 |  | 1.54 (0.94 to 2.51) | 0.147 |
| *P*_interaction_ |  |  |  |  |  | <0.001 |
| rs11849530 |  |  |  |  |  |  |
| Wild genotype (AA) | No | 144 | 110 |  | 1.00 (reference) |  |
| Variant genotypes (GA+GG) | No | 172 | 112 |  | 1.17 (0.74 to 1.86) | 0.529 |
| Wild genotype (AA) | Yes | 86 | 106 |  | 1.28 (0.79 to 2.08) | 0.450 |
| Variant genotypes (GA+GG) | Yes | 102 | 136 |  | 2.01 (1.25 to 3.22) | 0.020 |
| *P*_interaction_ |  |  |  |  |  | 0.538 |
| rs1256142 |  |  |  |  |  |  |
| Wild genotype (GG) | No | 70 | 22 |  | 1.00 (reference) |  |
| Variant genotypes (GA+AA) | No | 246 | 200 |  | 2.12 (1.13 to 4.01) | 0.060 |
| Wild genotype (GG) | Yes | 38 | 56 |  | 2.38 (1.08 to 5.22) | 0.078 |
| Variant genotypes (GA+AA) | Yes | 150 | 186 |  | 2.94 (1.54 to 5.62) | 0.008 |
| *P*_interaction_ |  |  |  |  |  | <0.001 |

Abbreviations: CHD = congenital heart diseases; *MTHFD1* = methylenetetrahydrofolate dehydrogenase 1; aOR = adjusted odds ratio; CI= confidence interval; SNPs = single nucleotide polymorphisms; FDR_*P* = the false discovery rate *P* value.

^a^ FDR*_P* < 0.1 was considered to indicate a statistically significant difference;

^b^ Adjusted for residence location, maternal education level, annual income in the past 1 year, history of adverse pregnancy outcomes, consanguineous marriage, history of congenital malformations in family, cold or fever in the periconceptional period, and personal lifestyle and habit in the periconceptional period including drinking alcohol, drinking tea, living near environmental pollution source, dyeing hair or perming and folate use.

**Table S5. Crossover analysis in assessing the interactive effects between the maternal polymorphisms of *MTHFD1* gene and passive smoking in the first trimester on the risk of CHD**

| SNPs | Passive smoking in the first trimester | No. of  Control | No. of  Case |  | Multivariate analysis | |
| --- | --- | --- | --- | --- | --- | --- |
|  |  |  |  |  | aOR (95%CI)^b^ | FDR_*P*^a^ |
| rs1950902 |  |  |  |  |  |  |
| Wild genotype (GG) | No | 56 | 22 |  | 1.00 (reference) |  |
| Variant genotypes (GA+AA) | No | 350 | 252 |  | 1.99 (1.01 to 3.91) | 0.047 |
| Wild genotype (GG) | Yes | 16 | 24 |  | 3.74 (1.34 to 10.44) | 0.012 |
| Variant genotypes (GA+AA) | Yes | 82 | 166 |  | 3.97 (1.93 to 8.17) | 0.000 |
| *P*_interaction_ |  |  |  |  |  | 0.800 |
| rs2236225 |  |  |  |  |  |  |
| Wild genotype (GG) | No | 262 | 182 |  | 1.00 (reference) |  |
| Variant genotypes (GA+AA) | No | 144 | 92 |  | 0.96 (0.64 to 1.44) | 0.834 |
| Wild genotype (GG) | Yes | 70 | 104 |  | 1.74 (1.10 to 2.74) | 0.043 |
| Variant genotypes (GA+AA) | Yes | 28 | 86 |  | 2.93 (1.64 to 5.24) | <0.001 |
| *P*_interaction_ |  |  |  |  |  | 0.026 |
| rs2236222 |  |  |  |  |  |  |
| Wild genotype (AA) | No | 260 | 132 |  | 1.00 (reference) |  |
| Variant genotypes (GA+GG) | No | 146 | 142 |  | 1.49 (1.00 to 2.21) | 0.090 |
| Wild genotype (AA) | Yes | 58 | 122 |  | 3.06 (1.92 to 4.90) | <0.001 |
| Variant genotypes (GA+GG) | Yes | 40 | 68 |  | 1.85 (1.05 to 3.27) | 0.069 |
| *P*_interaction_ |  |  |  |  |  | 0.011 |
| rs11849530 |  |  |  |  |  |  |
| Wild genotype (AA) | No | 182 | 132 |  | 1.00 (reference) |  |
| Variant genotypes (GA+GG) | No | 224 | 142 |  | 1.16 (0.78 to 1.73) | 0.505 |
| Wild genotype (AA) | Yes | 48 | 84 |  | 1.62 (0.96 to 2.75) | 0.111 |
| Variant genotypes (GA+GG) | Yes | 50 | 106 |  | 3.22 (1.91 to 5.45) | <0.001 |
| *P*_interaction_ |  |  |  |  |  | 0.207 |
| rs1256142 |  |  |  |  |  |  |
| Wild genotype (GG) | No | 88 | 38 |  | 1.00 (reference) |  |
| Variant genotypes (GA+AA) | No | 318 | 236 |  | 1.55 (0.93 to 2.59) | 0.124 |
| Wild genotype (GG) | Yes | 20 | 40 |  | 2.08 (0.93 to 4.67) | 0.111 |
| Variant genotypes (GA+AA) | Yes | 78 | 150 |  | 3.36 (1.88 to 5.99) | <0.001 |
| *P*_interaction_ |  |  |  |  |  | 0.014 |

Abbreviations: CHD = congenital heart diseases; *MTHFD1* = methylenetetrahydrofolate dehydrogenase 1; aOR = adjusted odds ratio; CI= confidence interval; SNPs = single nucleotide polymorphisms; FDR_*P* = the false discovery rate *P* value.

^a^ FDR*_P* < 0.1 was considered to indicate a statistically significant difference;

^b^Adjusted for residence location, maternal education level, annual income in the past 1 year, history of adverse pregnancy outcomes, consanguineous marriage, history of congenital malformations in family, cold or fever in the periconceptional period, and personal lifestyle and habit in the periconceptional period including drinking alcohol, drinking tea, living near environmental pollution source, dyeing hair or perming and folate use
